# A Longitudinal Description of the Health-Related Quality of Life Among Individuals at High Risk After SARS-CoV-2 Infection: A Dutch Multicenter Observational Cohort Study

**DOI:** 10.1093/ofid/ofaf055

**Published:** 2025-01-30

**Authors:** Magda Vergouwe, Emma Birnie, Sarah van Veelen, Jason J Biemond, Brent Appelman, Hessel Peters-Sengers, Godelieve J de Bree, Stephanie Popping, W Joost Wiersinga, TURN-COVID Study Group, Matthijs R A Welkers, Frans J van Ittersum, Maarten F Schim van der Loeff, Marije K Bomers, Marie José Kersten, Mette D Hazenberg, Jarom Heijmans, Marc van der Valk, E Marleen Kemper, Marcel van den Berge, Heidi S M Ammerlaan, Marvin A H Berrevoets, Robbert J van Alphen, Renée A Douma, Eliane M S Leyten, Cees van Nieuwkoop, Rob J van Marum, Frits R Rosendaal, Mark G J de Boer, Astrid M L Oude Lashof, Marit G A van Vonderen, Jiri F P Wagenaar, Janneke E Stalenhoef, Frank van de Veerdonk, Robert-Jan Hassing, Robin Soetekouw, Hazra S Moeniralam, Frits van Osch

**Affiliations:** Center for Infection and Molecular Medicine, Amsterdam University Medical Center, Location AMC, University of Amsterdam, Amsterdam, the Netherlands; Amsterdam institute for Immunology and Infectious Diseases, Infectious Diseases, Amsterdam, the Netherlands; Center for Infection and Molecular Medicine, Amsterdam University Medical Center, Location AMC, University of Amsterdam, Amsterdam, the Netherlands; Amsterdam institute for Immunology and Infectious Diseases, Infectious Diseases, Amsterdam, the Netherlands; Division of Infectious Diseases, Department of Medicine, Amsterdam University Medical Center, University of Amsterdam, Amsterdam, the Netherlands; Center for Infection and Molecular Medicine, Amsterdam University Medical Center, Location AMC, University of Amsterdam, Amsterdam, the Netherlands; Center for Infection and Molecular Medicine, Amsterdam University Medical Center, Location AMC, University of Amsterdam, Amsterdam, the Netherlands; Amsterdam institute for Immunology and Infectious Diseases, Infectious Diseases, Amsterdam, the Netherlands; Center for Infection and Molecular Medicine, Amsterdam University Medical Center, Location AMC, University of Amsterdam, Amsterdam, the Netherlands; Amsterdam institute for Immunology and Infectious Diseases, Infectious Diseases, Amsterdam, the Netherlands; Center for Infection and Molecular Medicine, Amsterdam University Medical Center, Location AMC, University of Amsterdam, Amsterdam, the Netherlands; Department of Epidemiology and Data Science, Amsterdam University Medical Center, Location Vrije Universiteit Amsterdam, Amsterdam, the Netherlands; Amsterdam institute for Immunology and Infectious Diseases, Infectious Diseases, Amsterdam, the Netherlands; Division of Infectious Diseases, Department of Medicine, Amsterdam University Medical Center, University of Amsterdam, Amsterdam, the Netherlands; Center for Infection and Molecular Medicine, Amsterdam University Medical Center, Location AMC, University of Amsterdam, Amsterdam, the Netherlands; Amsterdam institute for Immunology and Infectious Diseases, Infectious Diseases, Amsterdam, the Netherlands; Department of Medical Microbiology and Infection Prevention, Amsterdam University Medical Center, University of Amsterdam, Amsterdam, the Netherlands; Center for Infection and Molecular Medicine, Amsterdam University Medical Center, Location AMC, University of Amsterdam, Amsterdam, the Netherlands; Amsterdam institute for Immunology and Infectious Diseases, Infectious Diseases, Amsterdam, the Netherlands; Division of Infectious Diseases, Department of Medicine, Amsterdam University Medical Center, University of Amsterdam, Amsterdam, the Netherlands

**Keywords:** COVID-19, health-related quality of life, HRQoL utility score, immunocompromised, SARS-CoV-2

## Abstract

**Background:**

Health-related quality of life (HRQoL) data post–COVID-19 in patients with medical conditions associated with severe disease are lacking. Here, we assess the longitudinal impact of COVID-19 on HRQoL and employment status in individuals at high risk.

**Methods:**

This multicenter prospective cohort study included individuals at high risk for severe disease who were hospitalized or not-hospitalized with SARS-CoV-2 infection (September 2021–February 2024). Questionnaires about HRQoL and employment status were collected at 3, 6, and 12 months post–COVID-19 and retrospectively recalled and reported for pre–COVID-19. With a mixed effects model, we assessed the course of and risk factors for changes in HRQoL utility score.

**Results:**

Among 332 individuals (median age, 59.8 years [IQR, 48.8–67.1]; 50.6% female), 184 (55.4%) were hospitalized for COVID-19 (intensive care unit admission, 12.0%). High-risk factors included solid organ transplantation (19.6%), hematologic malignancies (28.0%), and immunosuppressive medication use (56.6%). The median HRQoL utility score declined from 0.85 (IQR, 0.74–1.00) pre–COVID-19 to 0.81 (0.70–0.92) 12 months post–COVID-19 (*P* = .007). Solid organ transplant recipients and patients requiring oxygen therapy were at risk for an HRQoL decrease over 1 year. At 12 months, 45.3% of all employed responders had reported sick leave related to COVID-19 symptoms. Employed patients who reported sick leave had lower median HRQoL utility scores (0.81 [IQR, 0.72–0.91]) than those who did not (0.89 [0.86–1.00], *P* = .002).

**Conclusions:**

Solid organ transplant recipients and individuals requiring oxygen therapy experience a substantial HRQoL decline over 12 months post–COVID-19. Moreover, almost half of employed participants reported COVID-19–related sick leave, correlating with lower HRQoL. This highlights the continuous burden of COVID-19 for this vulnerable population and supports the implementation of preventive approaches.

## Teaser

Solid organ transplant recipients and hospitalized high-risk individuals requiring oxygen therapy experience a substantial decline in health-related quality of life over 12 months post–COVID-19. We underline the importance of preventive approaches and highlight the continuous disease burden of COVID-19 for this vulnerable patient population.

Since its emergence in December 2019, SARS-CoV-2 has heavily affected public health, including >775 million people to date [1, 2]. Although vaccines reduced morbidity and mortality, patients with certain preexisting medical conditions (eg, immunosuppressive) remain vulnerable to severe disease [3, 4]. Moreover, numerous individuals face persistent symptoms after COVID-19, including but not limited to dyspnea, fatigue, muscle weakness, and cognitive impairment [5–7]. The term *long COVID* is commonly used when symptoms persist >3 months [8]. The World Health Organization estimates the prevalence of long COVID at around 10% to 20% in all people infected by SARS-CoV-2 [7]. In a significant part of these patients, long-term conditions affect daily functioning, physical activities, work, and social life [9]. Studies in hospitalized patients with COVID-19 have shown substantial health-related quality of life (HRQoL) impairment over 12 months after acute infection, which is particularly pronounced in patients who are critically ill [10, 11]. This is highlighted by a recent report noting that only 69% of patients fully returned to work 12 months following COVID-19 hospitalization [12]. For nonhospitalized patients, studies on post–COVID-19 HRQoL have presented conflicting results. Some studies have demonstrated HRQoL burden [13], while others have reported a recovery to population norms [10].

The US Centers for Disease Control and Prevention identified key risk factors for severe COVID-19, including immunocompromising conditions such as solid organ transplantation and malignancies and chronic conditions such as obesity and diabetes [14]. Several of these comorbidities also increase the likelihood of developing long COVID, with an odds ratio of 1.50 (95% CI, 1.05–2.15) for immunosuppression [15]. Overall, individuals with chronic conditions report a 15%–71% lower HRQoL as compared with the general Dutch population [16–18]. Combined with their higher vulnerability to severe disease manifestations and long COVID, this potentially elevates the risk of impaired HRQoL after COVID-19. Furthermore, the management of COVID-19 in patients at high risk often involves costly and, in some regions, scarce COVID-19 preventative and therapeutic measures, including monoclonal antibodies and antiviral agents. Utility scores derived from real-world HRQoL data in this population, which are currently lacking, can be translated to quality-adjusted life-years, providing valuable input for cost-effectiveness analyses.

In this study, we aim to evaluate long-term physical and mental health outcomes, HRQoL, employment status, and risk factors for impaired HRQoL in individuals at high risk who were hospitalized and not hospitalized over 12 months following COVID-19.

## METHODS

### Study Cohort

We present data from the ongoing multicenter prospective TURN-COVID study performed in 19 Dutch hospitals [19–21]. Adults were invited to participate in the study between September 2021 and February 2024 if they (1) received SARS-CoV-2 monoclonal antibodies or antiviral agents at any site or (2) were untreated individuals at the coordinating site who had high-risk factors for severe disease [14]. As part of routine COVID-19 care, eligible persons with SARS-CoV-2 infection received casirivimab-imdevimab or sotrovimab between September 2021 and April 2022 and nirmatrelvir/ritonavir from November 2022 onward. During these periods, the Dutch SARS-CoV-2 viral landscape was characterized by the dominance of the Delta variant, followed by the Omicron BA.1, BA.2, BA.5, BQ.1, XBB, and JN.1 variants [22, 23].

### Study Design and Data Collection

Baseline and clinical characteristics were obtained from electronic health records. HRQoL was assessed by the EuroQol 5-Dimension 5-Level (EQ-5D-5L) questionnaire, and the Fatigue Severity Scale (FSS) and Hospital Anxiety and Depression Scale (HADS) were used to assess symptoms influencing specific HRQoL dimensions. The EQ-5D-5L was sent out during COVID-19 and at 3, 6, and 12 months post–COVID-19 and retrospectively recalled and reported for pre–COVID-19. The HADS and FSS were collected at 3 and 6 months and retrospectively recalled and reported for pre–COVID-19. COVID-19 symptoms were assessed during COVID-19 and at 3 and 6 months. Additionally, pre–COVID-19 and 6- and 12-month questionnaires inquired about employment status and the 12-month questionnaire about reinfection (Supplementary Table 1). Only participants who completed pre–COVID-19 and at least 1 follow-up questionnaire were included, excluding those who died within 3 months. This study conformed to STROBE guidelines (Strengthening the Reporting of Observational Studies in Epidemiology; Supplementary Appendix).

### HRQoL Questionnaires

#### EQ-5D-5L Questionnaire

The EQ-5D-5L is a validated HRQoL instrument measuring HRQoL utility scores focusing on 5 dimensions of quality of life: mobility, self-care, usual activities, pain/discomfort, and anxiety/depression [24]. Each dimension is rated on 5 severity levels (no, slight, moderate, severe, and extreme). The combination of dimensions and severity levels results in a health state, which was converted into a HRQoL utility score by the Dutch EQ-5D-5L value set [16]. Scores range from 1 (perfect health) to 0 (death), with negative values indicating worse-than-death health states. This questionnaire has been validated for the Dutch population and language [25]. Furthermore, the questionnaire incorporates the EQ visual analog scale (VAS), a patient's self-reported current health status on a scale from best (100) to worst (0) [24].

#### FSS and HADS Questionnaires

The FSS is a validated self-reported measure on the interference of fatigue with certain activities, comprising 9 statements rated on a 7-point Likert-type scale [26]. We assessed the FSS by determining the average score across the 9 items, where a score ≥4 indicates clinically significant fatigue. The HADS is a validated self-reported questionnaire used to evaluate anxiety and depression and consists of 14 items divided into 2 subscales: anxiety and depression [27]. Both subscales comprise 7 items presented in alternating order, and statements can be answered by a 4-point Likert-type scale. Consequently, subscores range from 0 to 21, where outcomes ≥11 represent abnormal scores and 8 to 10 borderline abnormal. Publicly available Dutch versions were used for both questionnaires [28].

### Statistical Analyses

Differences in baseline characteristics between groups (oxygen therapy groups and patients lost to follow-up vs others) were calculated by 1-way analysis of variance, Kruskal-Wallis test, χ^2^ test, unpaired *t* test, or Mann-Whitney *U* test as appropriate. Correlation between the EQ-5D-5L and the FSS and HADS was assessed by Spearman rank correlation. HRQoL utility and VAS score changes were assessed with mixed linear models (*nlme* package version 3.1-162). In the mixed models, time (before and during COVID-19 and 3, 6, and 12 months after) was incorporated as a factor predictor variable to account for nonlinear relationships over time and patient identification as a random intercept to correct for repeated measurements. Model assumptions were tested by assessing histograms and the skewness and kurtosis of the residuals. To meet model assumptions, the HRQoL utility score was transformed by the Box-Cox method. Changes in binary outcomes were analyzed over time with logistic mixed models (*lme4* package version 1.1-34). Again, patient identification was incorporated as a random intercept and time as a factor predictor variable. When model conversion problems occurred, we simplified the model using the *bobyqa* optimizer. Differences between patients lost and not lost to follow-up at 1 year post–COVID-19 are described in Supplementary Table 2.

To identify factors influencing the HRQoL utility score and its slope from pre–COVID-19 to 12 months, relevant variables were incorporated into a linear mixed model on pre–COVID-19 and 12-month HRQoL utility scores (*nlme* package version 3.1-162). Given the nonlinear course of the HRQoL utility score over time and our interest in HRQoL change over 1 year, we excluded HRQoL data points during and 3 and 6 months after COVID-19. Each variable with expected clinical relevance based on literature was tested in a separate mixed effects model to assess its fixed effect and interaction (Supplementary Table 3). The final model included (1) as fixed effects, either variables with univariate correlation to the HRQoL utility score with significance or those with high clinical relevance without significance and (2) as interaction terms, variables with significant interactions with time. We provide 95% CIs around estimates, with corresponding *P* values from the final adjusted models. To validate our results, we conducted a sensitivity analysis using the same model including HRQoL utility scores from pre–COVID-19 and 3, 6, and 12 months after. All statistical analyses were performed with R Studio version 4.2.1. *P* < .05 defined statistical significance.

### Patient Consent Statement

The study was reviewed by the Amsterdam UMC Research Ethics Committee (W21_383 #21.425 and NL78705.018.21) and applied under the non-WMO (Medical Research Involving Human Subjects Act). All participants provided written informed consent. The TURN-COVID study is registered at ClinicalTrials.gov (NCT05195060).

## RESULTS

### Patient Characteristics

Of 1604 individuals considered eligible for inclusion, 332 were included in the present study (Supplementary Figure 1). Follow-up questionnaires were available from 85 participants during COVID-19 and from 291, 264, and 191 at 3, 6, and 12 months post–COVID-19, respectively. The mean (SD) age was 57.6 (14.2) years, 168 (50.6%) were female, and 299 (90.1%) originated from Western Europe (Table 1, Supplementary Table 4). The majority of the population completed lower secondary or vocational schooling. Comorbidities associated with higher risk for severe COVID-19 were prevalent: obesity (body mass index >30 kg/m^2^; n = 67, 20.2%), chronic obstructive pulmonary disease (n = 32, 9.6%), hematologic malignancy (n = 93, 28.0%), solid organ transplant recipient (n = 65, 19.6%), and maintenance immunosuppressive medication (n = 188, 56.6%). The majority of participants (n = 209, 63.0%) received at least 1 SARS-CoV-2 vaccination. Hospitalization was required for 184 people (55.4%), of which 40 (21.7%) were admitted to the intensive care unit. Oxygen was administered to 162 patients (48.8%): 82 (50.6%) with low-flow oxygen and 80 (49.4%) with high-flow oxygen or invasive ventilation. We report no 90-day mortality, as our analysis solely included participants who completed questionnaires at or after 3 months. Differences in baseline characteristics between patients with and without 1-year HRQoL follow-up are presented in Supplementary Table 2. Notably, the prevalence of hematologic malignancies was lower in patients with 1-year follow-up (19.9%) as compared with patients without (39.0%). At 3 months, 200 of 291 (68.7%) had at least 1 symptom and at 6 months 180 of 264 (68.2%). The most prevalent symptoms at 6 months were fatigue (n = 134, 50.8%), muscle or joint pain (n = 70, 26.5%), coughing (n = 49, 18.6%), and dyspnea (n = 45, 17.0%). Among 191 patients completing the 12-month questionnaire, 55 (28.8%) had a reinfection at a median 238 days (IQR, 132–307) after the initial infection.

**Table 1.** Baseline Characteristics and Outcomes of Individuals at High Risk With SARS-CoV-2 Infection (N = 322)

Characteristic	No. (%)
**Demographics**	
Age, y, mean ± SD	57.6 ± 14.2
Sex	
Female	168 (50.6)
Male	164 (49.4)
Body mass index, kg/m^2^, mean ± SD^a^	27.0 ± 4.9
**Clinical characteristics**	
Received at least 1 SARS-CoV-2 vaccination	209 (63.0)
SARS-CoV-2 variant^b^	
Delta	172 (51.8)
Omicron	160 (48.2)
COVID-19–specific treatment	312 (94.0)
Neutralizing SARS-CoV-2 monoclonal antibodies^c^	275 (82.8)
Antiviral agents^d^	39 (11.7)
IL-6 receptor antagonists	60 (18.1)
Corticosteroids	154 (46.4)
**Comorbidities**	
Charlson Comorbidity Index, median (IQR)	3.0 (1.0–4.0)
Obesity (body mass index ≥30 kg/m^2^)^a^	67 (20.2)
Cardiovascular disease^e^	76 (22.9)
Medicated hypertension	81 (24.4)
Diabetes mellitus	46 (13.9)
Chronic kidney disease	57 (17.2)
Chronic obstructive pulmonary disease	32 (9.6)
Hematologic malignancy	93 (28.0)
Solid malignancy	13 (3.9)
Solid organ transplant^f^	65 (19.6)
Rheumatic disease	65 (19.6)
Primary immunodeficiency	9 (2.7)
**Immunosuppressive medication**	
Active immunosuppressive medication^g^	188 (56.6)
Corticosteroids	125 (37.7)
B- or T-cell inhibitors	145 (43.7)
Chemotherapy	16 (4.8)
Other^h^	40 (12.0)
**Outcomes**	
Hospitalized for COVID-19	184 (55.4)
Oxygen therapy^i^	
Low flow	82 (24.7)
High flow/invasive ventilation	80 (24.1)
Intensive care unit admission	40 (12.0)
Length of hospital stay, d, median (IQR)	8.0 (4.0–14.0)

Data are presented as No. (%) unless noted otherwise.

^a^Missing values in 49 patients.

^b^Variant of infection determined by the dominant variant in the Netherlands at the time of positive SARS-CoV-2 polymerase chain reaction result [19, 20].

^c^Casirivimab/imdevimab in 191 patients, sotrovimab in 83, and tixagevimab/cilgavimab in 1.

^d^Nirmatrelvir/ritonavir in 38 patient and remdesivir in 1.

^e^Including chronic heart disease, peripheral vascular disease, and cerebrovascular disease.

^f^Kidney in 48 patients, lung in 12, liver in 3, kidney and lung in 1, and small intestine in 1.

^g^Therapies prescribed in the outpatient care setting before study inclusion.

^h^Including hydroxycarbamide, hydroxychloroquine, methotrexate, lenalidomide, adalimumab, bevacizumab, etanercept, infliximab, pomalidomide, ruxolitinib, sulfasalazine, tofacitinib, and ustekinumab.

^i^Low-flow systems include nasal oxygen cannula, nasal catheter, and mouth-nose mask up to 6 L/min. High-flow systems include high-flow nasal cannula, Optiflow, Vapotherm, Venturi mask, and non-rebreathing mask >6 L/min.

### Longitudinal Changes in HRQoL Utility Scores

**Figure 1.**
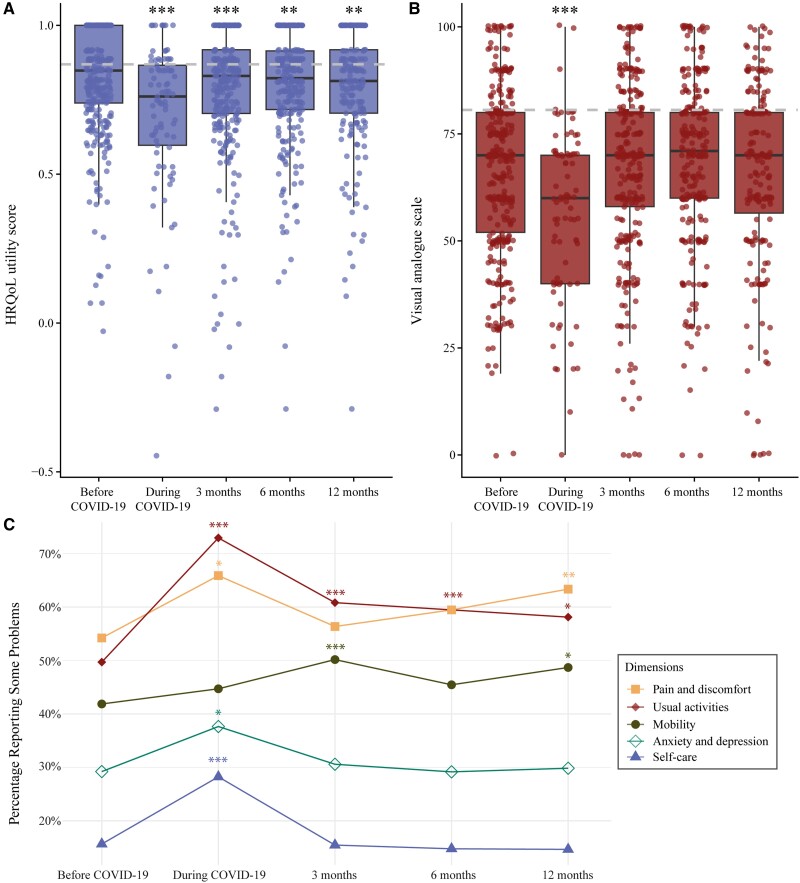
Change from pre–COVID-19 to COVID-19 and 3, 6, and 12 month post–COVID-19: *A*, EQ-5D-5L HRQoL utility scores; *B*, VAS scores; *C*, percentages reporting problems in dimensions pain and discomfort, usual activities, mobility, anxiety and depression, and usual activities. HRQoL utility and VAS scores are reported as medians. *A* and *B*, The gray striped lines represent the mean reference values of the general population in the Netherlands. Bold line, median; box, IQR; whiskers, 1.5× IQR. *P* values are derived from (*A* and *B*) linear and (*C*) logistic mixed models. *P < .05. **P < .01. *** *P* < .001. EQ-5D-5L, EuroQol 5-Dimension 5-Level; HRQoL, health-related quality of life; VAS, visual analog scale.

We analyzed HRQoL utility score changes over time up to 1 year post–COVID-19 (Figure 1). The median HRQoL utility score pre–COVID-19 was 0.85 (IQR, 0.74–1.00). During SARS-CoV-2 infection, the HRQoL utility score significantly declined to 0.76 (IQR, 0.60–0.87; *P* < .001). Post–COVID-19, the HRQoL utility score improved yet remained significantly lower than pre–COVID-19 at 3 months (0.83; IQR, 0.72–0.91; *P* < .001), 6 months (0.82; IQR, 0.72–0.91; *P* = .009), and 12 months (0.81; IQR, 0.70–0.92; *P* = .007). The HRQoL utility scores at all follow-up points for several subgroups are provided in Supplementary Table 5. The median HRQoL utility score at 12 months post–COVID-19 was similar for patients reporting reinfection (0.79; IQR, 0.72–0.96) vs those without reinfection (0.82; IQR, 0.70–0.92; *P* = .733). The mean (SD) VAS score significantly declined from 66.4 (19.2) pre–COVID-19 to 56.8 (20.0) during COVID-19 (*P* < .001; Figure 1*B*). However, after 3 months, the VAS scores returned to pre–COVID-19 levels (66.8 [20.4]) and remained stable over 6 months (68.7 [19.2]) and 12 months (65.8 [21.7]).

**Figure 2.**
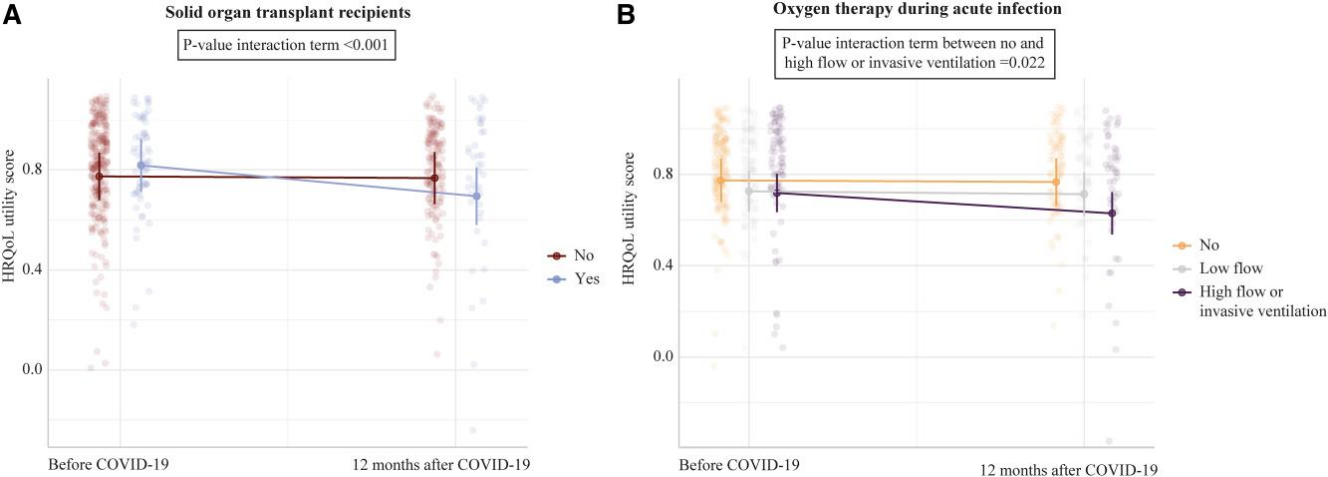
Predicted HRQoL utility score values of significant predictors of HRQoL decrease from pre–SARS-CoV-2 infection to 1 year after. *A*, The dark dots indicate the estimated HRQoL utility scores for solid organ transplant recipients (blue, n = 65) and patients without a solid organ transplant (red, n = 267) pre–COVID-19 and 12 months after COVID-19; the vertical lines represent the 95% CI of the estimate. The light shaded dots represent the true data. *B*, The dark dots indicate the estimated HRQoL utility scores for patients without oxygen therapy (yellow, n = 170), with low-flow oxygen (grey, n = 82), and with high-flow oxygen or invasive ventilation (purple, n = 80); the vertical lines represent the 95% CI of the estimate. The light shaded dots represent the true data. A linear mixed effect model was used to provide *P* values. HRQoL, health-related quality of life.

To identify variables influencing the HRQoL utility score, we conducted a mixed effects model (Supplementary Table 3, Table 2). Two variables significantly interacted with the HRQoL utility score course from pre–COVID-19 to 1 year post–COVID-19 (Figure 2). The decrease in the HRQoL utility score over 1 year for patients treated with high-flow oxygen or invasive ventilation was relatively higher as compared with patients without oxygen therapy (*P* = .022). Differences in baseline characteristics of oxygen therapy groups are provided in Supplementary Table 6. Among underlying medical conditions associated with higher risk for severe COVID-19, solid organ transplant recipient status was significantly associated with a relative decrease in HRQoL utility scores over 1 year (*P* < .001). We found associations between overall HRQoL utility scores and sex (estimate, −0.07; 95% CI, −.10 to −.03; *P* < .001), vaccination status (estimate, −0.07; 95% CI, −.12 to −.02; *P* = .012), diabetes (estimate, −0.06; 95% CI, −.12 to .00; *P* = .041), and education level (estimate, 0.16; 95% CI, .08–.24; *P* < .001). The sensitivity analysis including data pre–COVID-19 and 3, 6, and 12 months post–COVID-19 yielded similar results for the fixed effects and the interactions (Supplementary Table 7).

**Table 2.** Mixed Effects Model on Variables Influencing the Course of the HRQoL Utility Score Over 12 Months After SARS-CoV-2 Infection

	Coefficient (95% CI)	*P* Value
**Fixed effects**		
Age	0.001 (.000, .003)	.314
Sex^a^	−0.066 (−.104, −.027)	<.001
Moment^b^	−0.007 (−.070, .057)	.838
SARS-CoV-2 vaccination^c^	−0.069 (−.123, −.016)	.012
Solid organ transplant recipient^d^	0.044 (−.015, .103)	.147
Hematologic malignancy^e^	−0.010 (−.067, .048)	.737
Obesity^f^	0.004 (−.049, .056)	.892
Diabetes^g^	−0.061 (−.120, −.003)	.041
Charlson Comorbidity Index	−0.010 (−.025, .006)	.223
Education^h^	0.159 (.081, .238)	<.001
Oxygen therapy^i^		
Low-flow oxygen therapy	−0.048 (−.103, .008)	.094
High-flow oxygen therapy	−0.055 (−.117, .008)	.086
**Interactions**		
Moment: vaccinated^c^	0.046 (−.015, .107)	.143
Moment: solid organ transplant recipient^d^	−0.116 (−.181, −.051)	<.001
Moment: hematologic malignancy^e^	0.006 (−.051, .070)	.854
Moment: obesity^f^	−0.046 (−.106, .014)	.135
Moment: oxygen therapy^i^		
Moment: oxygen low flow	−0.007 (−.067, .055)	.850
Moment: oxygen high flow	−0.083 (−.153, −.012)	.022

The coefficients portray the estimated increases or decreases in HRQoL utility scores for each variable. HRQoL utility score data pre–COVID-19 and 12 months post–COVID-19 were included in this model.

Abbreviations: HRQoL, health-related quality of life.

^a^Reference category is male sex.

^b^Pre–COVID-19 and 1 year post–COVID-19. The reference category is pre–COVID-19.

^c^Reference category is no SARS-CoV-2 vaccination.

^d^Reference category is no solid organ transplant.

^e^Reference category is no hematologic malignancy.

^f^Defined as body mass index ≥30 kg/m^2^. Reference category is no obesity.

^g^Reference category is no diabetes.

^h^Reference category is no education.

^i^Reference category is no oxygen therapy.

### Persistent Problems in EQ-5D-5L Dimensions: Mobility, Usual Activities, and Pain and Discomfort

The proportion of individuals reporting problems in 4 dimensions of the EQ-5D-5L significantly increased during SARS-CoV-2 infection vs pre–COVID-19: self-care (28.2% vs 15.7%, *P* < .001), usual activities (72.9% vs 49.7%, *P* < .001), anxiety and depression (37.6% vs 29.2%, *P* = .040), and pain and discomfort (65.9% vs 54.2%, *P* = .019; Figure 1*C*, Supplementary Figure 2). Problems in 3 dimensions remained significantly higher after 12 months as compared with pre–COVID-19: mobility (48.7% vs 41.9%, *P* = .015), usual activities (58.1% vs 49.7%, *P* = .011), and pain and discomfort (63.4% vs 54.2%, *P* = .007).

### Significant Fatigue and Depression-Related Symptoms 6 Months Post–COVID-19

**Figure 3.**
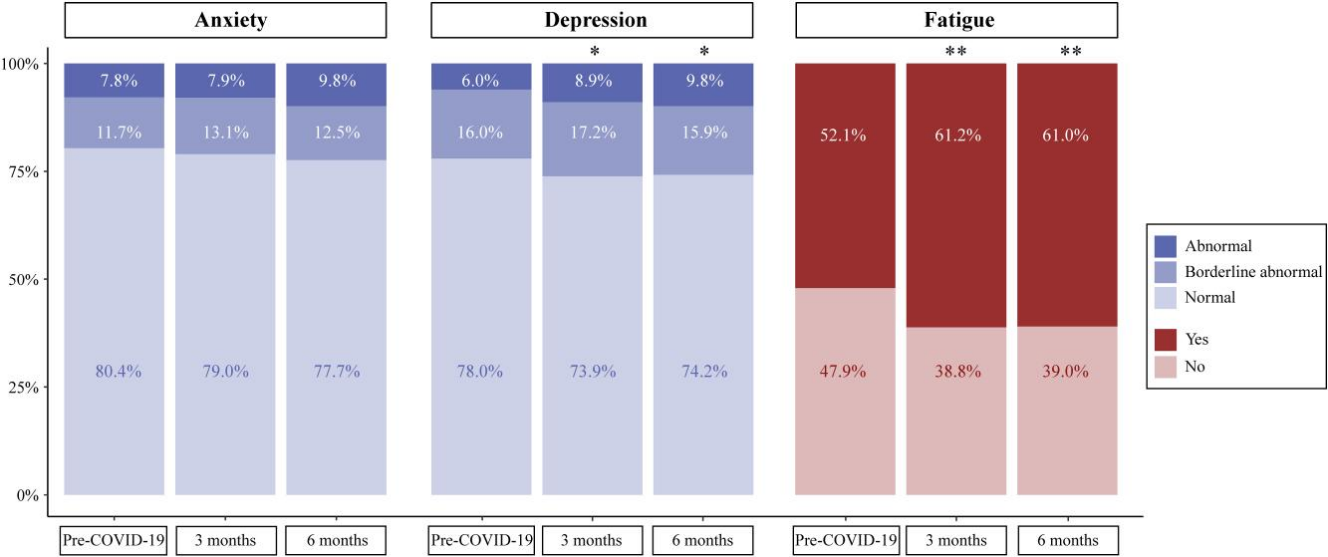
Self-reported outcomes of the Hospital Anxiety and Depression Scale and Fatigue Severity Scale pre–COVID-19 and 3 and 6 months post–COVID-19. The percentage of patients with abnormal scores on the Hospital Anxiety and Depression Scale or a positive fatigue score on the Fatigue Severity Scale at 3 and 6 months was compared with the percentage before COVID-19 with logistic mixed models. *P < .05. **P < .01.

Abnormal HADS scores pre–COVID-19 were found in 6.0% (depression) and 7.8% (anxiety) of patients (Figure 3). Three and 6 months after COVID-19, abnormal depression scores increased to 8.9% (*P* = .023) and 9.8% (*P* = .024), respectively. For anxiety, no significant change was observed after 3 (7.9% abnormal) or 6 (9.8% abnormal) months. The EQ-5D-5L anxiety and depression dimension significantly correlated with the depression (ρ = 0.48, *P* < .001) and anxiety (ρ = 0.59, *P* < .001) HADS subscales.

Fatigue was evaluated by the FSS. Pre–COVID-19, 52.1% of participants had an average FSS score ≥4, indicative of clinically significant fatigue (Figure 3). The percentage of patients with fatigue significantly increased to 61.2% after 3 months (*P* = .002) and 61.0% after 6 months (*P* = .001). The FSS outcomes significantly correlated to the EQ-5D-5L usual activities dimension (ρ = 0.57, *P* < .001) and HRQoL utility score (ρ = −0.59, *P* < .001).

### Employment Status

Pre–COVID-19, 135 (40.7%) participants had a paid job, 88 (26.5%) were retired, and 63 (19.0%) reported work disability. After 6 months, 99 of 251 (39.4%) had a paid job and after 12 months 75 of 191 (39.3%). Of those employed at 12 months, 34 (45.3%) were sick due to COVID-19 between 6 and 12 months. At 12 months, employed patients who took sick leave had significantly lower median (IQR) HRQoL utility scores as compared with those who did not take sick leave (0.81 [0.72–0.91] vs 0.89 [0.86–1.00], *P* = .002).

## DISCUSSION

To the best of our knowledge, this is the first study examining HRQoL among individuals at high risk 1 year after SARS-CoV-2 Delta and Omicron infection. HRQoL decreased substantially during COVID-19, which partially improved but remained below pre–COVID-19 levels 1 year post–COVID-19. Three HRQoL dimensions were particularly affected: mobility, usual activities, and pain and discomfort. Also, we report a significant increase in depression and fatigue-related symptoms at 3 and 6 months post–COVID-19. Patients requiring high-flow oxygen or invasive ventilation during SARS-CoV-2 infection and solid organ transplant recipients were identified as being at risk for a decline in HRQoL from pre–COVID-19 to 12 months post–COVID-19. Moreover, a substantial number of participants reported COVID-19–related sick leave the year following infection.

The mean (SD) pre–COVID-19 HRQoL utility score in our cohort (0.82 [0.19]) is substantially lower than the Dutch population norm of 0.87 (0.17). Since we included patients at high risk with lower pre–COVID-19 values, we chose to focus on the change within our cohort rather than comparing it with the general population or other study cohorts. We report a significant 0.04-point decrease in HRQoL utility score, with a median 0.81 (IQR, 0.70–0.92) and mean (SD) 0.79 (0.20) at 12 months. Consensus about the minimum clinically important difference for utility scores in cohorts after infectious disease is lacking, but a review showed ranges from 0.03 to 0.52 varying across disease contexts [29]. Given these ranges, we cannot rule out a clinically significant difference, particularly for patients with larger declines. For example, in solid organ transplant recipients, a decline of 0.06 points has been observed, suggesting that changes are more significant for specific subgroups.

Solid organ transplant recipients had a relatively larger HRQoL decline post–COVID-19 as compared with other patients, irrespective of hospitalization status. While COVID-19 outcomes have been explored extensively in this subpopulation, long-term HRQoL studies are lacking. Our results highlight the vulnerability of solid organ transplant recipients and the potential value of implementing preventative or early therapeutic measures, such as nirmatrelvir/ritonavir, in this group to alleviate the impact on HRQoL and yield cost-saving benefits [20]. Of note, we did not find associations between decline in HRQoL over 1 year and other immunocompromising conditions, such as hematologic malignancies. This might reflect follow-up bias, as we report a substantially lower frequency of hematologic malignancies in patients with 1-year follow-up (19.9%) as compared with those without (39.0%; Supplementary Table 2). Further research on HRQoL post–COVID-19 in patients with hematologic malignancy is crucial to assess the burden and identify at-risk subpopulations.

Apart from solid organ transplant recipients, HRQoL was significantly reduced among those who required high-flow oxygen or invasive ventilation during acute infection, aligning with prior studies [10, 11, 30]. As illustrated in Supplementary Table 5, 90% of our cohort requiring high-flow oxygen or invasive ventilation had the Delta variant. Therefore, we cannot state whether a lower HRQoL in hospitalized patients after Omicron infection is to be expected. Despite the lower odds of long COVID after Omicron infection vs prior variants, the risk remains substantial [7]. Continued viral evolution could result in a more pathogenic variant, increasing hospitalization rates [31].

To assess HRQoL, we used the generic EQ-5D-5L questionnaire. As the EQ-5D-5L is extensively used among several medical fields, it is considered a robust and reliable method. Furthermore, its extensive validation, brevity, ease of administration and interpretation, and ability to compare with the general population make it a useful tool for this study. However, a generic instrument may lack in-depth evaluation of COVID-19–specific or dimension-specific changes. For example, we did not find a significant increase in problems in the anxiety and depression dimension of the EQ-5D-5L but did identify significantly more patients exhibiting abnormal depression scores at 3 and 6 months when using the HADS. Therefore, we encourage the application of more in-depth questionnaires as an addition to the EQ-5D-5L.

Our data showed a stable reduced HRQoL from 3 to 12 months post–COVID-19 with several dimensions affected, which could be explained by ongoing symptoms. We hypothesize that this loss may persist, which is supported by recent data showing lower HRQoL up to 3 years postinfection among patients exhibiting long COVID symptoms 2 years postinfection [32]. Consequently, increased long-term health care utilization can be expected. Implementing early physical and mental rehabilitation or guidance posthospitalization may help prevent long-term burden [33].

Strikingly, nearly half of employed participants had sick leave related to COVID-19 symptoms between 6 and 12 months postinfection. Additionally, we found lower HRQoL in patients who had reported sick leave, aligning with prior studies [12, 30]. High sick leave rates potentially affect society's productivity but also patients and their families financially and their well-being overall.

Our data provide valuable input for cost-effectiveness analyses evaluating future, often costly, therapies in high-risk populations, as the HRQoL utility values can be used to calculate quality-adjusted life years. Currently, HRQoL utility values in cost-effectiveness analyses are often derived by modeling, vignette-based values, or other respiratory diseases [34, 35]. These methods have limitations and do not reflect the health status of actual patients. Continuously collecting longitudinal real-world HRQoL data provides a robust framework for cost-effectiveness analyses in this vulnerable population, which is expected to remain at risk for SARS-CoV-2 infections.

Our study has several strengths. First, we focus on patients who are vulnerable and at high risk for severe disease. This group is currently understudied with respect to the long-term impact of SARS-CoV-2 infection on HRQoL; moreover, it is a vital group to track as it represents the target population for therapies such as nirmatrelvir/ritonavir. Second, the pre–COVID-19 HRQoL was compared with that of post–COVID-19, which provided a more individualized approach to understanding COVID-19's impact on HRQoL. In addition to the generic HRQoL measure EQ-5D-5L, we used the FSS and HADS to provide a more in-depth assessment of specific dimensions of HRQoL. Moreover, we included hospitalized and nonhospitalized cases, ensuring a more representative and applicable interpretation of our results in diverse clinical contexts among a high-risk population.

Yet, certain study limitations should be considered. First, pre–COVID-19 questionnaires were filled out retrospectively, possibly introducing recall bias. However, prior research proved high agreement between retrospective and prospective EQ-5D-5L measurements [36]. Second, our cohort is heterogenic, encompassing different SARS-CoV-2 variants and comorbidities, which posed challenges in generalizing the findings. We acknowledge the possibility that the observed HRQoL decrease may be attributed to the underlying comorbidities rather than solely to COVID-19. We attempted to mitigate this confounding effect by incorporating comorbidities into our mixed model analysis. Last, our data may be an underestimation of the true HRQoL burden, as dropouts among sicker patients and missing data of deceased individuals were not taken into consideration.

In conclusion, our study showed the enduring HRQoL impact of COVID-19 among solid organ transplant recipients and those with high-flow oxygen requirements. The sustained reduction in HRQoL and impact on work status underline the long-term disease burden faced by this vulnerable population. We emphasize that in addition to public health measures such as vaccination and hygiene practices for the general population, exploring and implementing strategies such as antiviral agents and monoclonal antibodies remains crucial for individuals at high risk to mitigate the prolonged consequences of COVID-19. Furthermore, this study provides valuable insights and real-world data for future cost-effectiveness analyses.

## Supplementary Material

ofaf055_Supplementary_Data

## Supplementary Data

Supplementary materials are available at *Open Forum Infectious Diseases* online. Consisting of data provided by the authors to benefit the reader, the posted materials are not copyedited and are the sole responsibility of the authors, so questions or comments should be addressed to the corresponding author.

## Notes

***Acknowledgments*.** We thank the research nurses and clinicians at the Amsterdam University Medical Center and all the patients who participated in this study. The research presented is part of a Dutch cohort study on neutralizing monoclonal antibodies and other antiviral agents against SARS-CoV-2 (TURN-COVID; NCT05195060). We thank the following members of the TURN-COVID study group who were not otherwise compensated: Amsterdam University Medical Centers, Amsterdam (Matthijs R. A. Welkers, Frans J. van Ittersum, Maarten F. Schim van der Loeff, Marije K. Bomers, Marie José Kersten, Mette D. Hazenberg, Jarom Heijmans, Marc van der Valk); Dutch Hospital Pharmacists’ Association (NVZA; E. Marleen Kemper); Admiraal de Ruyter Hospital, Goes (Marcel van den Berge); Catharina Hospital, Eindhoven (Heidi S. M. Ammerlaan); Elisabeth TweeSteden Hospital, Tilburg (Marvin A. H. Berrevoets, Robbert J. van Alphen); Flevohospital, Almere (Renée A. Douma); Haaglanden Medical Center (Eliane M. S. Leyten); Haga Teaching Hospital, The Hague (Cees van Nieuwkoop); Jeroen Bosch Hospital (Rob J. van Marum); Leiden University Medical Center, Leiden (Frits R. Rosendaal, Mark G. J. de Boer); Maastricht University Medical Center (Astrid M. L. Oude Lashof); Medical Centre Leeuwarden, Leeuwarden (Marit G. A. van Vonderen); Northwest Clinics Alkmaar (Jiri F. P. Wagenaar); OLVG Hospital, Amsterdam (Janneke E. Stalenhoef); Radboud University Medical Center (Frank van de Veerdonk); Rijnstate Hospital (Robert-Jan Hassing); Spaarne Gasthuis, Haarlem (Robin Soetekouw); St. Antonius Hospital (Hazra S. Moeniralam); VieCuri Medical Center, Venlo (Frits van Osch).

***Author contributions*.** Conceptualization: W. J. W., S. P., E. B., G. J. B. Data collection and data curation: M. V., E. B., S. V. V., J. J. B., B. A., H. P.-S., G. J. B., S. P., W. J. W. Data analysis: M. V., S. V. V., H. P.-S., S. P. Drafting the manuscript: M. V., E. B., S. V. V., W. J. W., S. P. Reviewing and editing the manuscript: all authors. Supervision: S. P., E. B., W. J. W. Obtained funding: W. J. W., E. B.

***Disclaimer.*** The funders had no role in the design and conduct of the study; collection, management, analysis, and interpretation of the data; preparation, review, or approval of the manuscript; and decision to submit the manuscript for publication.

***Financial support.*** This work was supported by the Netherlands Organization for Health Research and Development (ZonMw; TURN-COVID study 10430142110001).
