# Supplementary material for: A Longitudinal Description of the Health-Related Quality of Life Among Individuals at High Risk After SARS-CoV-2 Infection: A Dutch Multicenter Observational Cohort Study
Source: Open Forum Infect Dis. 2025 Jan 30;12(2):ofaf055. doi: 10.1093/ofid/ofaf055 (PMC11837172; doi:10.1093/ofid/ofaf055)
Supplement: ofaf055_Supplementary_Data [file ofaf055_supplementary_data.docx]

**Supplementary Appendix**

**A longitudinal description of the health-related quality of life among high-risk individuals after SARS-CoV-2 infection: a Dutch multicenter observational cohort study**

Magda Vergouwe^1,2^, Emma Birnie^1,2,3^, Sarah van Veelen^1^, Jason J. Biemond^1,2^, Brent Appelman^1,2^, Hessel Peters-Sengers^1,4^, Godelieve J. de Bree^2,3^, Stephanie Popping^1,2,5*^, W. Joost Wiersinga^1,2,3*^ on behalf of the TURN-COVID Study group^#^

^*^ Both senior authors contributed equally

^#^ Study group members are listed in the Acknowledgements

^1^ Center for Infection and Molecular Medicine (CIMM), Amsterdam University Medical Center, University of Amsterdam, Amsterdam, the Netherlands

^2^ Amsterdam institute for Immunology and Infectious Diseases, Infectious Diseases, Amsterdam, the Netherlands

^3^ Division of Infectious Diseases, Department of Medicine, Amsterdam University Medical Center, University of Amsterdam, Amsterdam, the Netherlands

^4^ Department of Epidemiology and Data Science, Amsterdam University Medical Center, Vrije Universiteit Amsterdam, Amsterdam, The Netherlands

^5^ Department of Medical Microbiology and Infection Prevention, Amsterdam University Medical Center, University of Amsterdam, Amsterdam, the Netherlands

**Corresponding author**: Stephanie Popping, MD, PhD, Amsterdam UMC - Location AMC, University of Amsterdam, Center for Infection and Molecular Medicine, Meibergdreef 9, Amsterdam, Netherlands; Phone number: +31205669111; Email: [s.popping@amsterdamumc.nl](mailto:s.popping@amsterdamumc.nl)

**Table of Content**

[**METHODS** 3](#_Toc184306598)

[**Table S1.** Overview of surveys and data collection at study follow-up points 3](#_Toc184306599)

[**Table S2.** Baseline characteristics and outcomes of patients with and without one-year HRQoL follow-up available 4](#_Toc184306600)

[**Table S3.** Overview of tested and selected variables for model inclusion 5](#_Toc184306601)

[**RESULTS** 6](#_Toc184306602)

[**Figure S1.** Flowchart **of the inclusion process of the study** 6](#_Toc184306603)

[**Table S4.** Ethnicity, employment and education data of included patients 7](#_Toc184306604)

[**Table S5.** HRQoL_utility_ scores for clinical subgroups at all follow-up points 8](#_Toc184306605)

[**Table S6.** Baseline characteristics and outcomes per oxygen therapy group 9](#_Toc184306606)

[**Table S7.** Mixed**-**effects model on variables influencing the health-related quality of life (HRQoL) utility score course from pre-COVID-19 to 3, 6 and 12 months after COVID-19. 10](#_Toc184306607)

[**Figure S2.** The levels of problems perceived per dimension mobility, self-care, usual activities, pain and discomfort, and anxiety and depression from pre-COVID-19 to during COVID-19 and three, six, and 12 months after COVID-19 11](#_Toc184306608)

[**REFERENCES** 12](#_Toc184306609)

[**STROBE (Strengthening the Reporting of Observational Studies in Epidemiology) Statement**  13](#_Toc184306610)

# **METHODS**

|  | **Before COVID-19** | **During COVID-19** | **+ 3 months** | **+ 6 months** | **+ 12 months** |
| --- | --- | --- | --- | --- | --- |
| **Symptoms** |  | x | x | x |  |
| **Reinfection** |  |  |  |  | x |
| **Working status** | x |  | x | x | x |
| **EQ-5D-5L** | x | x | x | x | x |
| **HADS** | x |  | x | x |  |
| **FSS** | x |  | x | x |  |

## **Table S1. Overview of surveys and data collection at study follow-up points**

Abbreviations: EQ-5D-5L: EuroQol 5-dimension 5-level questionnaire; HADS: Hospital Anxiety and Depression Scale; FSS: Fatigue Severity Scale.

| **Characteristic** | **Patients without one year HRQoL follow-up** | **Patients with one year HRQoL follow-up** |
| --- | --- | --- |
| **No** (%) | 141 | 191 |
| **Demographics** |  |  |
| Age, years, mean ± SD | 58.5 ± 15.4 | 56.9 ± 13.2 |
| Sex |  |  |
| Female | 74 (52.5) | 94 (49.2) |
| Male | 67 (47.5) | 97 (50.8) |
| Body Mass Index, kg/m^2^, mean ± SD^1^ | 26.5 ± 5.1 | 27.4 ± 4.8 |
| **Clinical characteristics** |  |  |
| Received at least one SARS-CoV-2 vaccination | 94 (66.7) | 115 (60.2) |
| SARS-CoV-2 variant ^2^ |  |  |
| Delta | 58 (41.1) | 114 (59.7) |
| Omicron | 83 (58.9) | 77 (40.3) |
| COVID-19-specific treatment |  |  |
| Neutralizing SARS-CoV-2 monoclonal antibodies | 91 (64.5) | 184 (96.3) |
| Antiviral agents | 38 (27.0) | 1 (0.5) |
| IL-6 receptor antagonist | 20 (14.2) | 40 (20.9) |
| Corticosteroids | 59 (41.8) | 95 (49.7) |
| **Comorbidities** |  |  |
| Charlson Comorbidity Index, median (IQR) | 3.0 (2.0-5.0) | 3.0 (1.0-4.0) |
| Obesity (Body Mass Index ≥ 30 kg/m2) | 27 (19.1) | 40 (20.9) |
| Cardiovascular disease ^3^ | 32 (22.7) | 44 (23.0) |
| Medicated hypertension | 36 (25.5) | 45 (23.6) |
| Diabetes mellitus | 17 (12.1) | 29 (15.2) |
| Chronic kidney disease | 24 (17.0) | 33 (17.3) |
| Chronic obstructive pulmonary disease | 15 (10.6) | 17 (8.9) |
| Hematologic malignancy | 55 (39.0) | 38 (19.9) |
| Solid malignancy | 4 (2.8) | 9 (4.7) |
| Solid organ transplant | 23 (16.3) | 42 (22.0) |
| Rheumatic disease | 23 (16.3) | 42 (22.0) |
| Primary immunodeficiency | 6 (4.3) | 3 (1.6) |
| **Immunosuppressive medication** |  |  |
| Active immunosuppressive medication ^4^ | 78 (55.3) | 110 (57.6) |
| Corticosteroids | 52 (36.9) | 73 (38.2) |
| B- or T- cell inhibitors | 55 (39.0) | 90 (47.1) |
| Chemotherapy | 6 (4.3) | 10 (5.2) |
| Other ^5^ | 16 (11.3) | 24 (12.6) |
| **Outcomes** |  |  |
| Hospitalized for COVID-19 | 72 (51.1) | 112 (58.6) |
| Oxygen therapy ^6^ |  |  |
| Low-flow | 30 (21.3) | 52 (27.2) |
| High-flow/invasive ventilation | 33 (23.4) | 47 (24.6) |
| Intensive Care Unit admission | 14 (9.9) | 26 (13.6) |
| Length of hospital stay, days, median (IQR) | 9.0 (4.0-14.3) | 8.0 (4.0-14.0) |

## **Table S2. Baseline characteristics and outcomes of patients with and without one-year HRQoL follow-up available**

Abbreviations: SD: standard deviation; SARS-CoV-2: Severe acute respiratory syndrome coronavirus 2; COVID-19: Coronavirus disease 2019; IQR: interquartile range.

^1^ Missing values in 49 patients;

^2^ Variant of infection determined by the dominant variant in the Netherlands at the time of positive SARS-CoV-2 PCR [1,2].

^3^ Including chronic heart disease, peripheral vascular disease, and cerebrovascular disease;

^4^ Therapies prescribed in the outpatient care setting before study inclusion;

^5^ Including Hydroxycarbamide, hydroxychloroquine, methotrexate, lenalidomide, adalimumab, bevacizumab, etanercept, infliximab, pomalidomide, ruxolitinib, sulfasalazine, tofacitinib and ustekinumab.

^6^ Low-flow systems include nasal oxygen cannula, nasal catheter, mouth-nose mask up to 6 L/min. High-flow systems include high flow nasal cannula (HFNC), Optiflow, Vapotherm, Venturi mask and non-rebreathing mask over 6 L/min.

| **Variables tested** | **Fixed effect *P* < 0.05** | **Interaction *P* < 0.05** | **Variable included in final model** |
| --- | --- | --- | --- |
| Age | No | No | Fixed effect (included based on clinical relevance) |
| Sex | Yes | No | Fixed effect |
| Education level | Yes | No | Fixed effect |
| Vaccination status | No | Yes | Fixed effect and interaction term |
| SARS-CoV-2 variant | No | No | Not included |
| Charlson comorbidity index | Yes | No | Fixed effect |
| Obesity (Body Mass Index ≥ 30 kg/m^2^) | No | Yes | Fixed effect and interaction term |
| Chronic obstructive pulmonary disease | No | No | Not included |
| Diabetes | Yes | No | Fixed effect |
| Medicated hypertension | No | No | Not included |
| Solid organ transplantation | No | Yes | Fixed effect and interaction term |
| Hematologic malignancy | No | Yes | Fixed effect and interaction term |
| Immunocompromised other^1^ | No | No | Not included |
| Reinfection within 12 months | No | No | Not included |
| Oxygen therapy | No | Yes | Fixed effect and interaction term |
|  |  |  |  |

## **Table S3. Overview of tested and selected variables for model inclusion**

To identify factors influencing HRQoL_utility_ scores and the slope of HRQoL_utility_ scores from pre-COVID-19 to 12 months, relevant variables were incorporated in a linear mixed model on HRQoL_utility_ scores data pre-COVID-19 and at 12 months. Each variable with expected clinical relevance based on literature was tested for univariate significance in a mixed model with HRQoL_utility_ score as the outcome, as well as for significant interactions with the time variable. Variables that showed univariate significance with the HRQoL_utility_ score or high clinical relevance without significance were included as fixed effects in the final model. Variables with significant interactions with time were incorporated as interaction terms in the final model.

^1^ Patients without solid organ transplantation or hematologic malignancy who are immunocompromised based on immunosuppressive medication use or a primary immunodeficiency disorder.

# **RESULTS**


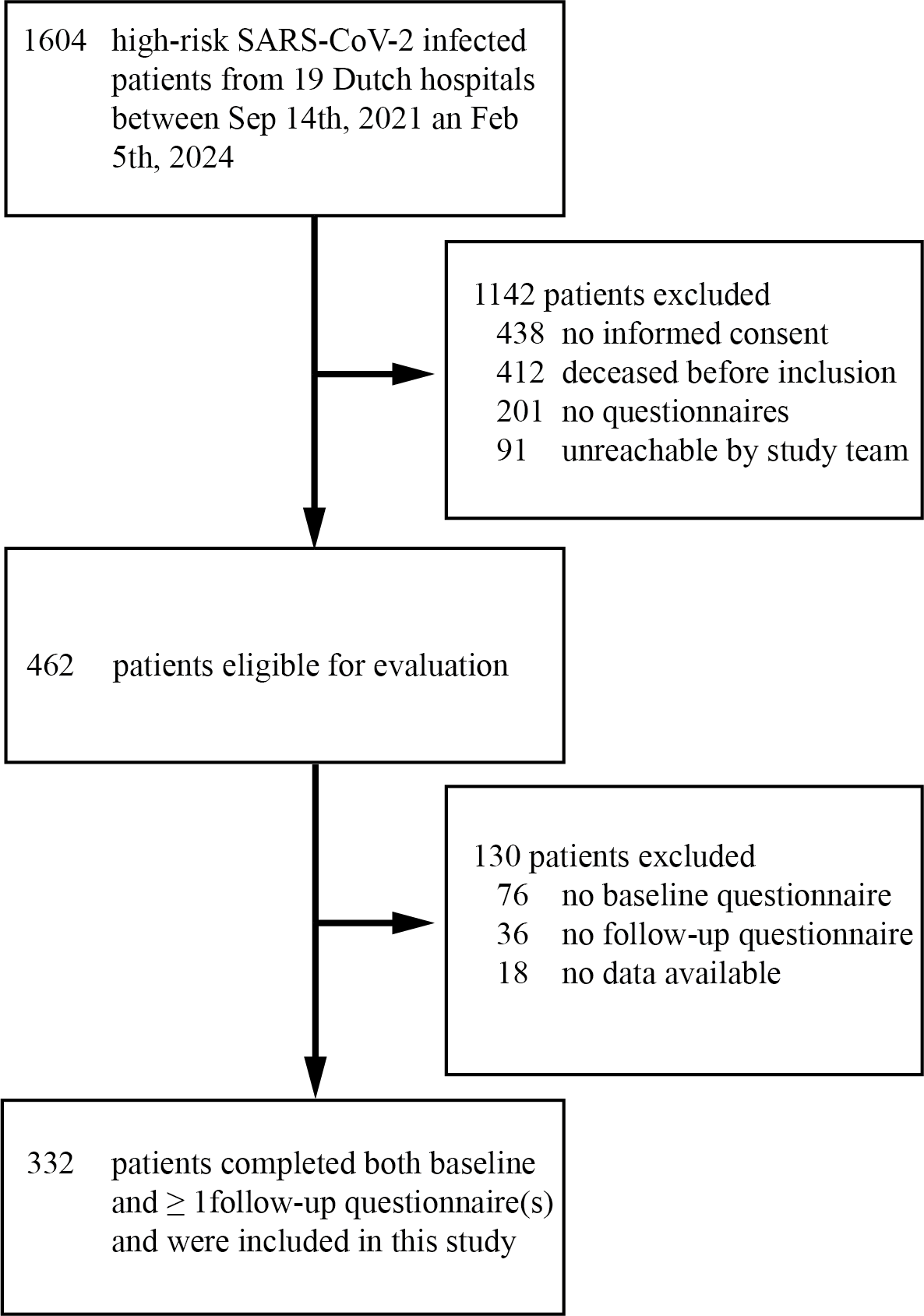


## **Figure S1.** Flowchart representing the inclusion process of the present study. All patients treated with monoclonal antibodies or antiviral agents for a SARS-CoV-2 infection in 19 Dutch hospitals alive at inclusion were considered eligible for inclusion. Patients who completed the baseline and at least one follow-up questionnaire were included in the present study. Abbreviations: SARS-CoV-2: Severe acute respiratory syndrome coronavirus 2.

| **Number of patients, no** | 332 |
| --- | --- |
| **Region country of birth** |  |
| Western Europe | 299 (90.1) |
| Surinam and Netherlands Antilles | 14 (4.2) |
| Other | 19 (5.7) |
| **Employment (baseline)** |  |
| Employed | 135 (40.7) |
| Retired | 88 (26.5) |
| Work disability | 63 (19.0) |
| Homemaker | 26 (7.8) |
| Unemployed | 13 (3.9) |
| Studying | 7 (2.1) |
| **Education** |  |
| Lower secondary or vocational schooling | 161 (48.5) |
| Intermediate/higher secondary schooling | 102 (30.7) |
| University | 48 (14.5) |
| No education or elementary schooling | 21 (6.3) |

## **Table S4. Ethnicity, employment and education data of included patients**

|  | **Before COVID-19** | **n (%)** |  | **During COVID-19** | **n (%)** |  | **3 months post-COVID-19** | **n (%)** |  | **6 months post-COVID-19** | **n (%)** |  | **12 months post-COVID-19** | **n (%)** |
| --- | --- | --- | --- | --- | --- | --- | --- | --- | --- | --- | --- | --- | --- | --- |
| **HRQoL_utility_** |  |  |  |  |  |  |  |  |  |  |  |  |  |  |
| Median (IQR) | 0.85 (0.74-1.00) | 332 (100) |  | 0.76 (0.60-0.87) | 85 (100) |  | 0.83 (0.70-0.92) | 291 (100) |  | 0.82 (0.72-0.91) | 264 (100) |  | 0.81 (0.70-0.92) | 191 (100) |
| Mean ± SD | 0.82 ± 0.19 | 332 (100) |  | 0.69 ± 0.27 | 85 (100) |  | 0.78 ± 0.22 | 291 (100) |  | 0.79 ± 0.19 | 264 (100) |  | 0.79 ± 0.20 | 191 (100) |
| Sex |  |  |  |  |  |  |  |  |  |  |  |  |  |  |
| Female | 0.81 (0.71-1.00) | 168 (50.6) |  | 0.76 (0.53-0.88) | 40 (47.1) |  | 0.79 (0.67-0.88) | 147 (50.5) |  | 0.80 (0.70-0.89) | 132 (50.0) |  | 0.79 (0.70-0.89) | 94 (49.2) |
| Male | 0.88 (0.77-1.00) | 164 (49.4) |  | 0.75 (0.61-0.85) | 45 (52.9) |  | 0.85 (0.74-1.00) | 144 (49.5) |  | 0.86 (0.77-1.00) | 132 (50.0) |  | 0.85 (0.74-1.00) | 97 (50.8) |
| Age, years |  |  |  |  |  |  |  |  |  |  |  |  |  |  |
| < 40 | 0.85 (0.74-1.00) | 43 (13.0) |  | 0.77 (0.53-0.88) | 13 (15.3) |  | 0.84 (0.70-1.00) | 40 (13.7) |  | 0.83 (0.74-0.92) | 34 (12.9) |  | 0.80 (0.70-0.89) | 21 (11.0) |
| 40-60 | 0.85 (0.76-1.00) | 125 (37.7) |  | 0.85 (0.78-0.89) | 26 (30.6) |  | 0.81 (0.73-0.92) | 104 (35.7) |  | 0.85 (0.74-1.00) | 103 (39.0) |  | 0.81 (0.74-1.00) | 83 (43.5) |
| > 60 | 0.82 (0.69-1.00) | 164 (49.4) |  | 0.71 (0.58-0.84) | 46 (54.1) |  | 0.83 (0.69-0.91) | 147 (50.5) |  | 0.81 (0.71-0.89) | 127 (48.1) |  | 0.82 (0.70-0.92) | 87 (45.5) |
| Education |  |  |  |  |  |  |  |  |  |  |  |  |  |  |
| No | 0.68 (0.60-0.85) | 21 (6.3) |  | 0.66 (0.64-0.74) | 3 (3.5) |  | 0.71 (0.65-0.90) | 17 (5.8) |  | 0.75 (0.57-0.84) | 16 (6.1) |  | 0.74 (0.62-0.92) | 9 (4.7) |
| Low secondary/vocational | 0.85 (0.75-1.00) | 161 (48.5) |  | 0.75 (0.52-0.88) | 30 (35.3) |  | 0.80 (0.68-0.88) | 140 (48.1) |  | 0.81 (0.70-0.89) | 125 (47.3) |  | 0.81 (0.72-0.91) | 95 (49.7) |
| Intermediate/higher secondary | 0.82 (0.74-1.00) | 102 (30.7) |  | 0.75 (0.59-0.89) | 30 (35.3) |  | 0.83 (0.73-1.00) | 90 (30.9) |  | 0.85 (0.74-0.91) | 82 (31.1) |  | 0.81 (0.68-1.00) | 60 (31.4) |
| University | 0.85 (0.74-1.00) | 48 (14.5) |  | 0.78 (0.64-0.85) | 22 (25.9) |  | 0.89 (0.81-1.00) | 44 (15.1) |  | 0.89 (0.81-1.00) | 41 (15.5) |  | 0.88 (0.69-1.00) | 27 (14.1) |
| Solid organ transplantation |  |  |  |  |  |  |  |  |  |  |  |  |  |  |
| Yes | 0.85 (0.75-1.00) | 65 (19.6) |  | 0.83 (0.63-0.88) | 15 (17.6) |  | 0.83 (0.70-1.00) | 59 (20.3) |  | 0.85 (0.71-1.00) | 52 (19.7) |  | 0.79 (0.67-0.98) | 42 (22.0) |
| No | 0.85 (0.73-1.00) | 267 (80.4) |  | 0.74 (0.60-0.86) | 70 (82.4) |  | 0.83 (0.70-0.91) | 232 (79.7) |  | 0.82 (0.72-0.91) | 212 (80.3) |  | 0.83 (0.72-0.92) | 149 (78.0) |
| Hospital admission |  |  |  |  |  |  |  |  |  |  |  |  |  |  |
| Yes | 0.82 (0.71-1.00) | 184 (55.4) |  | 0.65 (0.46-0.76) | 28 (32.9) |  | 0.80 (0.65-0.88) | 154 (52.9) |  | 0.81 (0.70-0.90) | 146 (55.3) |  | 0.81 (0.70-0.91) | 112 (58.6) |
| No | 0.85 (0.75-1.00) | 148 (44.6) |  | 0.80 (0.67-0.88) | 57 (67.1) |  | 0.86 (0.76-1.00) | 137 (47.1) |  | 0.86 (0.75-1.00) | 118 (44.7) |  | 0.85 (0.73-1.00) | 79 (41.4) |
| Oxygen therapy^1^ |  |  |  |  |  |  |  |  |  |  |  |  |  |  |
| None | 0.85 (0.74-1.00) | 170 (51.2) |  | 0.80 (0.63-0.88) | 69 (81.2) |  | 0.85 (0.76-1.00) | 154 (52.9) |  | 0.86 (0.74-1.00) | 133 (50.4) |  | 0.83 (0.74-1.00) | 92 (48.2) |
| Low Flow | 0.81 (0.68-1.00) | 82 (24.7) |  | 0.66 (0.56-0.73) | 12 (14.1) |  | 0.78 (0.67-0.85) | 69 (23.7) |  | 0.78 (0.65-0.89) | 64 (24.2) |  | 0.82 (0.70-0.94) | 52 (27.2) |
| High Flow/invasive ventilation | 0.85 (0.75-1.00) | 80 (24.1) |  | 0.43 (0.18-0.48) | 4 (4.7) |  | 0.80 (0.61-0.88) | 68 (23.4) |  | 0.80 (0.71-0.89) | 67 (25.4) |  | 0.81 (0.68-0.88) | 47 (24.6) |

## **Table S5. HRQoL_utility_ scores for clinical subgroups at all follow-up points**

Abbreviations: COVID-19: Coronavirus disease 2019; HRQoL: Health-relted quality of life; IQR: interquartile range; SD: standard deviation.

^1^ Low-flow systems include nasal oxygen cannula, nasal catheter, mouth-nose mask up to 6 L/min. High-flow systems include high flow nasal cannula (HFNC), Optiflow, Vapotherm, Venturi mask and non-rebreathing mask over 6 L/min.

|  | **No oxygen therapy** | **Low-flow oxygen^6^** | **High-flow oxygen^6^ or invasive ventilation** |
| --- | --- | --- | --- |
| **No (%)** | 170 | 82 | 80 |
| **Demographics** |  |  |  |
| Age, years, mean ± SD | 54.6 ± 14.0 | 62.3 ± 13.4 | 59.2 ± 13.9 |
| Sex |  |  |  |
| Female | 95 (55.9) | 42 (51.2) | 31 (38.8) |
| Male | 75 (44.1) | 40 (48.8) | 49 (61.2) |
| Body Mass Index, kg/m^2^, mean ± SD ^1^ | 25.9 ± 4.6 | 27.6 ± 5.2 | 28.5 ± 4.9 |
| **Clinical characteristics** |  |  |  |
| Received at least one SARS-CoV-2 vaccination | 146 (85.9) | 42 (51.2) | 21 (26.3) |
| SARS-CoV-2 variant ^2^ |  |  |  |
| Delta | 45 (26.5) | 55 (67.1) | 72 (90.0) |
| Omicron | 125 (73.5) | 27 (32.9) | 8 (10.0) |
| COVID-19-specific treatment |  |  |  |
| Neutralizing SARS-CoV-2 monoclonal antibodies | 126 (74.1) | 71 (86.6) | 78 (97.5) |
| Antiviral agents | 31 (18.2) | 8 (9.8) | 0 (0.0) |
| IL-6 receptor antagonists | 1 (0.6) | 15 (18.3) | 44 (55.0) |
| Corticosteroids | 3 (1.8) | 72 (87.8) | 79 (98.8) |
| **Comorbidities** |  |  |  |
| Charlson comorbidity index, median (IQR) | 3.0 (2.0-4.0) | 3.0 (1.3-5.0) | 2.0 (1.0-4.0) |
| Obesity (Body Mass Index ≥ 30 kg/m^2^) ^1^ | 20 (11.8) | 20 (24.4) | 27 (33.8) |
| Cardiovascular disease ^3^ | 29 (17.1) | 21 (25.6) | 26 (32.5) |
| Medicated hypertension | 35 (20.6) | 21 (25.6) | 25 (31.3) |
| Diabetes mellitus | 21 (12.4) | 11 (13.4) | 14 (17.5) |
| Chronic kidney disease | 32 (18.8) | 15 (18.3) | 10 (12.5) |
| Chronic obstructive pulmonary disease (COPD) | 10 (5.9) | 11 (13.4) | 11 (13.8) |
| Hematologic malignancy | 70 (41.2) | 16 (19.5) | 7 (8.8) |
| Solid malignancy | 3 (1.8) | 8 (9.8) | 2 (2.5) |
| Solid organ transplant | 43 (25.3) | 13 (15.9) | 9 (11.3) |
| Rheumatic disease | 45 (26.5) | 12 (14.6) | 8 (10.0) |
| Primary immunodeficiency | 9 (5.3) | 0 (0.0) | 0 (0.0) |
| **Immunosuppressive medication** |  |  |  |
| Active immunosuppressive medication ^4^ | 126 (74.1) | 42 (51.2) | 20 (25.0) |
| Corticosteroids | 77 (45.3) | 34 (41.5) | 14 (17.5) |
| B- or T- cell inhibitors | 106 (62.4) | 25 (30.5) | 14 (17.5) |
| Chemotherapy | 9 (5.3) | 4 (4.9) | 3 (3.8) |
| Other ^5^ | 25 (14.7) | 10 (12.2) | 5 (6.3) |
| **Outcomes** |  |  |  |
| Hospitalized for COVID-19 | 22 (12.9) | 82 (100.0) | 80 (100.0) |
| Intensive Care Unit admission | 0 (0.0) | 0 (0.0) | 40 (50.0) |
| Length of hospital stay, days, median (IQR) | 3.0 (2.0-5.0) | 6.0 (3.3-9.0) | 13.5 (8.0-21.3) |

## **Table S6. Baseline characteristics and outcomes per oxygen therapy group**

Abbreviations: SD: standard deviation; SARS-CoV-2: Severe acute respiratory syndrome coronavirus 2; COVID-19: Coronavirus disease 2019; IQR: interquartile range.

^1^ Missing values in 49 patients;

^2^ Variant of infection determined by the dominant variant in the Netherlands at the time of positive SARS-CoV-2 PCR [1,2].

^3^ Including chronic heart disease, peripheral vascular disease, and cerebrovascular disease;

^4^ Therapies prescribed in the outpatient care setting before study inclusion;

^5^ Including Hydroxycarbamide, hydroxychloroquine, methotrexate, lenalidomide, adalimumab, bevacizumab, etanercept, infliximab, pomalidomide, ruxolitinib, sulfasalazine, tofacitinib and ustekinumab.

^6^ Low-flow systems include nasal oxygen cannula, nasal catheter, mouth-nose mask up to 6 L/min. High-flow systems include high flow nasal cannula (HFNC), Optiflow, Vapotherm, Venturi mask and non-rebreathing mask over 6 L/min.

|  | **Coefficient (95%CI)** | ***P* value** |
| --- | --- | --- |
| **Fixed effects** |  |  |
| Age | 0.001 (0.000, 0.003) | 0.330 |
| Sex^1^ | -0.082 (-0.119, -0.046) | <0.001 |
| Moment^2^ |  |  |
| 3 months | -0.059 (-0.110, -0.008) | 0.023 |
| 6 months | -0.015 (-0.069, 0.038) | 0.575 |
| 12 months | 0.000 (-0.061, 0.062) | 0.996 |
| SARS-CoV-2 vaccination^3^ | -0.065 (-0.120, -0.010) | 0.021 |
| Solid organ transplant recipient^4^ | 0.047 (-0.014, 0.108) | 0.131 |
| Hematologic malignancy^5^ | -0.014 (-0.069, 0.048) | 0.726 |
| Obesity^6^ | 0.009 (-0.045, 0.062) | 0.760 |
| Diabetes^7^ | -0.071 (-0.127, -0.016) | 0.012 |
| Charlson comorbidity index | -0.012 (-0.026, 0.003) | 0.123 |
| Education^8^ | 0.121 (0.047, 0.194) | 0.002 |
| Oxygen therapy^9^ |  |  |
| Low flow oxygen therapy | -0.047 (-0.104, 0.009) | 0.103 |
| High flow oxygen therapy | -0.058 (-0.122, 0.005) | 0.074 |
|  |  |  |
| **Interactions** |  |  |
| Moment: Vaccinated^3^ |  |  |
| 3 months | 0.056 (0.006, 0.106) | 0.028 |
| 6 months | 0.029 (-0.023, 0.082) | 0.271 |
| 12 months | 0.037 (-0.022, 0.096) | 0.222 |
| Moment: Solid organ transplant recipient^4^ |  |  |
| 3 months | 0.002 (-0.053, 0.057) | 0.954 |
| 6 months | -0.022 (-0.080, 0.035) | 0.446 |
| 12 months | -0.107 (-0.170, -0.043) | 0.001 |
| Moment: Hematologic malignancy^5^ |  |  |
| 3 months | 0.034 (-0.015, 0.083) | 0.178 |
| 6 months | 0.008 (-0.044, 0.060) | 0.766 |
| 12 months | 0.020 (-0.042, 0.082) | 0.525 |
| Moment: Obesity^6^ |  |  |
| 3 months | -0.008 (-0.057, 0.041) | 0.753 |
| 6 months | 0.005 (-0.047, 0.057) | 0.839 |
| 12 months | -0.043 (-0.101, 0.015) | 0.146 |
| Moment: Oxygen low-flow^9^ |  |  |
| 3 months | -0.033 (-0.084, 0.018) | 0.202 |
| 6 months | -0.046 (-0.100, 0.007) | 0.090 |
| 12 months | -0.019 (-0.078, 0.040) | 0.529 |
| Moment: Oxygen high-flow^9^ |  |  |
| 3 months | -0.075 (-0.132, -0.018) | 0.010 |
| 6 months | -0.071 (-0.130, -0.011) | 0.020 |
| 12 months | -0.085 (-0.153, -0.017) | 0.014 |
|  |  |  |

## **Table S7. Mixed-effects model on variables influencing the Health-related quality of life (HRQoL) utility score course from pre-COVID-19 to 3, 6 and 12 months after COVID-19.**

The coefficients portray the estimated increases or decreases in HRQoL utility scores for each variable.
^1^ Reference category is male sex; ^2^ Pre-COVID-19 and one year post-COVID-19. The reference category is pre-COVID-19; ^3^ Reference category is no SARS-CoV-2 vaccination; ^4^ Reference category is no solid organ transplant; ^5^ Reference category is no hematologic malignancy; ^6^ Defined as Body Mass Index ≥ 30 kg/m^2^. Reference category is no obesity; ^7^ Reference category is no diabetes; ^8^ Reference category is no education; ^9^ Reference category is no oxygen therapy.


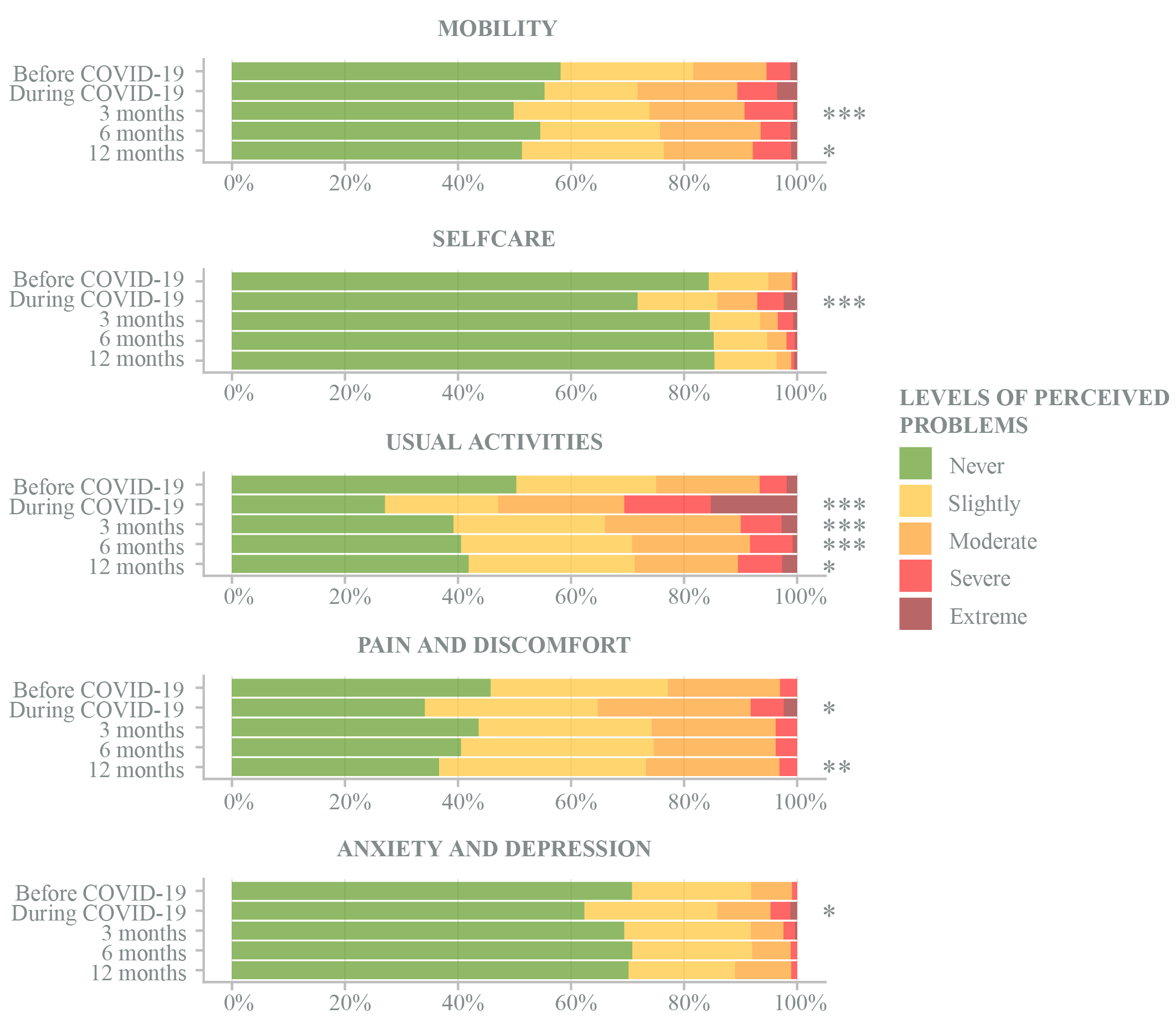


## **Figure S2.** The levels of problems perceived per dimension mobility, self-care, usual activities, pain and discomfort, and anxiety and depression from pre-COVID-19 to during COVID-19 and three, six, and 12 months after COVID-19. *P* values are derived from logistic mixed models. * *P* < 0.05, ** *P* < 0.01, *** *P* < 0.001.

# **REFERENCES**

1. RIVM. Variants of the coronavirus SARS-CoV-2. [https://www.rivm.nl/en/coronavirus-covid-19/current/variants. Accessed 17 June 2024](https://www.rivm.nl/en/coronavirus-covid-19/current/variants.%20Accessed%2017%20June%202024).
2. Slim MA, Appelman B, Peters-Sengers H, et al. Real-world Evidence of the Effects of Novel Treatments for COVID-19 on Mortality: A Nationwide Comparative Cohort Study of Hospitalized Patients in the First, Second, Third, and Fourth Waves in the Netherlands. *Open Forum Infect Dis* 2022; **9**: ofac632.

# **STROBE (Strengthening the Reporting of Observational Studies in Epidemiology) Statement - Checklist of items that should be included in reports of *cohort studies***

|  | Item No | Recommendation | Page number |
| --- | --- | --- | --- |
| **Title and abstract** | 1 | (*a*) Indicate the study’s design with a commonly used term in the title or the abstract | 1 |
|  |  | (*b*) Provide in the abstract an informative and balanced summary of what was done and what was found | 2 |
| Introduction | | |  |
| Background/rationale | 2 | Explain the scientific background and rationale for the investigation being reported | 3 |
| Objectives | 3 | State specific objectives, including any prespecified hypotheses | 3 |
| Methods | | |  |
| Study design | 4 | Present key elements of study design early in the paper | 4 |
| Setting | 5 | Describe the setting, locations, and relevant dates, including periods of recruitment, exposure, follow-up, and data collection | 4 |
| Participants | 6 | (*a*) Give the eligibility criteria, and the sources and methods of selection of participants. Describe methods of follow-up | 4 |
|  |  | (*b*) For matched studies, give matching criteria and number of exposed and unexposed | *NA* |
| Variables | 7 | Clearly define all outcomes, exposures, predictors, potential confounders, and effect modifiers. Give diagnostic criteria, if applicable | 4-5 |
| Data sources/ measurement | 8* | For each variable of interest, give sources of data and details of methods of assessment (measurement). Describe comparability of assessment methods if there is more than one group | 4-5 |
| Bias | 9 | Describe any efforts to address potential sources of bias | 5 |
| Study size | 10 | Explain how the study size was arrived at | 4 |
| Quantitative variables | 11 | Explain how quantitative variables were handled in the analyses. If applicable, describe which groupings were chosen and why | 5 |
| Statistical methods | 12 | (*a*) Describe all statistical methods, including those used to control for confounding | 5-6, and Appendix 5 |
|  |  | (*b*) Describe any methods used to examine subgroups and interactions | 5-6 |
|  |  | (*c*) Explain how missing data were addressed | 5 |
|  |  | (*d*) If applicable, explain how loss to follow-up was addressed | 5 |
|  |  | (*e*) Describe any sensitivity analyses | *NA* |
| Results | | |  |
| Participants | 13* | (a) Report numbers of individuals at each stage of study—eg numbers potentially eligible, examined for eligibility, confirmed eligible, included in the study, completing follow-up, and analysed | 6, Figure S1 |
|  |  | (b) Give reasons for non-participation at each stage | Figure S1 |
|  |  | (c) Consider use of a flow diagram | Figure S1 |
| Descriptive data | 14* | (a) Give characteristics of study participants (eg demographic, clinical, social) and information on exposures and potential confounders | 6-7 |
|  |  | (b) Indicate number of participants with missing data for each variable of interest | Table 1 |
|  |  | (c) Summarise follow-up time (eg, average and total amount) | 6 |
| Outcome data | 15* | Report numbers of outcome events or summary measures over time | 7-9 |
| Main results | 16 | (*a*) Give unadjusted estimates and, if applicable, confounder-adjusted estimates and their precision (eg, 95% confidence interval). Make clear which confounders were adjusted for and why they were included | 7-9 |
|  |  | (*b*) Report category boundaries when continuous variables were categorized | 8 |
|  |  | (*c*) If relevant, consider translating estimates of relative risk into absolute risk for a meaningful time period | *NA* |
| Other analyses | 17 | Report other analyses done—eg analyses of subgroups and interactions, and sensitivity analyses | *NA* |
| Discussion | | |  |
| Key results | 18 | Summarise key results with reference to study objectives | 9 |
| Limitations | 19 | Discuss limitations of the study, taking into account sources of potential bias or imprecision. Discuss both direction and magnitude of any potential bias | 10 |
| Interpretation | 20 | Give a cautious overall interpretation of results considering objectives, limitations, multiplicity of analyses, results from similar studies, and other relevant evidence | 9-11 |
| Generalisability | 21 | Discuss the generalisability (external validity) of the study results | 10 |
| Other information | | |  |
| Funding | 22 | Give the source of funding and the role of the funders for the present study and, if applicable, for the original study on which the present article is based | 12 |

*Give information separately for exposed and unexposed groups.

**Note:** An Explanation and Elaboration article discusses each checklist item and gives methodological background and published examples of transparent reporting. The STROBE checklist is best used in conjunction with this article (freely available on the Web sites of PLoS Medicine at http://www.plosmedicine.org/, Annals of Internal Medicine at http://www.annals.org/, and Epidemiology at http://www.epidem.com/). Information on the STROBE Initiative is available at http://www.strobe-statement.org.
